# Effects of Thickness and Amount of Carbon Nanofiber Coated Carbon Fiber on Improving the Mechanical Properties of Nanocomposites

**DOI:** 10.3390/nano6010006

**Published:** 2016-01-02

**Authors:** Ferial Ghaemi, Ali Ahmadian, Robiah Yunus, Fudziah Ismail, Saeed Rahmanian

**Affiliations:** 1 Institute of Tropical Forestry and Forest Products (INTROP), Universiti Putra Malaysia, 43400 UPM, Serdang, Selangor, Malaysia; robiah@upm.edu.my; 2 Department of Mathematics, Faculty of Sciences, and Institute for Mathematical Research (INSPEM), Universiti Putra Malaysia, 43400 UPM, Serdang, Selangor, Malaysia; ahmadian.hosseini@gmail.com (A.A.); fudziah@upm.edu.my (F.I.); 3 Department of Mechanical and Manufacturing Engineering, Universiti Putra Malaysia, 43400 UPM, Serdang, Selangor, Malaysia; saeedfed@gmail.com

**Keywords:** carbon fiber, carbon nanofiber, chemical vapor deposition, mechanical properties, polypropylene composite, mathematical model

## Abstract

In the current study, carbon nanofibers (CNFs) were grown on a carbon fiber (CF) surface by using the chemical vapor deposition method (CVD) and the influences of some parameters of the CVD method on improving the mechanical properties of a polypropylene (PP) composite were investigated. To obtain an optimum surface area, thickness, and yield of the CNFs, the parameters of the chemical vapor deposition (CVD) method, such as catalyst concentration, reaction temperature, reaction time, and hydrocarbon flow rate, were optimized. It was observed that the optimal surface area, thickness, and yield of the CNFs caused more adhesion of the fibers with the PP matrix, which enhanced the composite properties. Besides this, the effectiveness of reinforcement of fillers was fitted with a mathematical model obtaining good agreement between the experimental result and the theoretical prediction. By applying scanning electronic microscope (SEM), transmission electron microscope (TEM), and Raman spectroscopy, the surface morphology and structural information of the resultant CF-CNF were analyzed. Additionally, SEM images and a mechanical test of the composite with a proper layer of CNFs on the CF revealed not only a compactness effect but also the thickness and surface area roles of the CNF layers in improving the mechanical properties of the composites.

## 1. Introduction

Carbon fibers (CFs) with excellent properties, such as high strength and low weight have been used as fillers at a small percentage to reinforce polymer composites [1,2]. High interfacial adhesion between the polymer matrix and the CF provides a strong structure of the composites with effective load transfer from the polymer matrix to the CF. Besides this, various carbon nanomaterials can act as fillers in a polymer matrix [3,4,5]. Such nanomaterials with a variety of novel properties like, high specific modulus, strength, surface area, high chemical and thermal stability, low mass density, and high electric conductivity, have been widely studied in many fields of science and industry [5,6,7,8,9,10]. Hence, carbon nanofibers (CNFs) with high aspect ratios (length/diameter > 100) can be utilized as fillers in a polymer matrix to improve the mechanical and thermal properties of composites [11,12,13].

Additionally, the interaction of the CNF with the CF was reported to improve the interfacial adhesion between the fibers and the matrix of the polymer [14,15,16]. Therefore, growing the CNF on the CF fabricates a robust network in a polymer matrix [17].

To synthesis the carbon nanomaterials, many techniques, such as arc-discharge [6], laser ablation [7], chemical vapor deposition (CVD) [8,18], and coaxial electrospinning [19] have been employed. CVD as the most effective method, has been applied to grow the CNFs [20,21]. To achieve different structures and morphologies of the CNFs, some critical parameters of the CVD, such as growth time, growth temperature, flow rate of carbon source gas, and catalyst concentration can be varied [22,23,24,25,26].

In this study, different thicknesses of the CNF coated short carbon fiber reinforced polypropylene (CF-CNF/PP) were achieved and the theoretical prediction of the effective reinforcement of the fillers was calculated by a mathematical model. The mathematical model, named the Halpin–Tsai Model, has the most likely capability to compute the modulus of a composite material based on the filler content and the stiffness properties of the filler and matrix [27].

To the best of our knowledge, so far, nobody has investigated the simultaneous effects of surface area, thickness, and amount (yield) of the CNF layers coated with CFs as a filler on improving the mechanical properties of a polypropylene composite.

Consequently, in the current study, CNFs were synthesized on the surface of the CF using the CVD method under different process conditions (catalyst concentration, temperature, time, and hydrocarbon flow rate) and then they were used as fillers in a polypropylene matrix to fabricate the composite (CF-CNF/PP). Different types of CNF in aspects of surface area, thickness and amount (yield) were mixed with a PP matrix and compared regarding mechanical tests including the tensile test. The surface morphological and structural evolution of the CF-CNF was analyzed by scanning electron microscopy (SEM) and Raman spectroscopy.

## 2. Results and Discussion

### 2.1. Morphology of CNF

**Figure 1:**
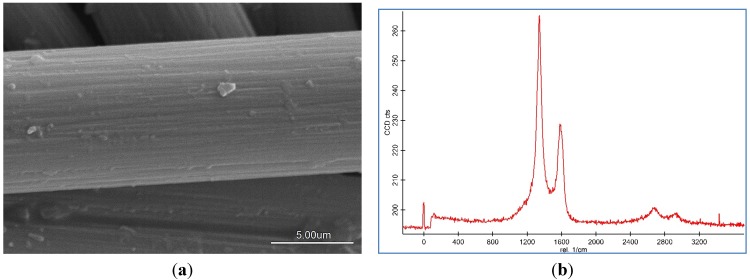
(**a**) Scanning electron microscopy (SEM) image; (**b**) Raman spectroscopy of pristine carbon fiber.

The morphology, population, thickness, distribution, and agglomeration of the CNF coated CF were demonstrated by SEM images. In addition, the structural information was evaluated by Raman spectroscopy. These features of CNF are dependent on various CVD process parameters. The SEM image and Raman spectra of pristine CF is revealed in Figure 1. The Brunauer–Emmett–Teller (BET) surface area of CF is about 0.71 m^2^/g. The tensile strength of CF is about 3800 MPa and its tensile modulus is 231 GPa.

### 2.2. Effect of Catalyst Concentration

**Figure 2:**
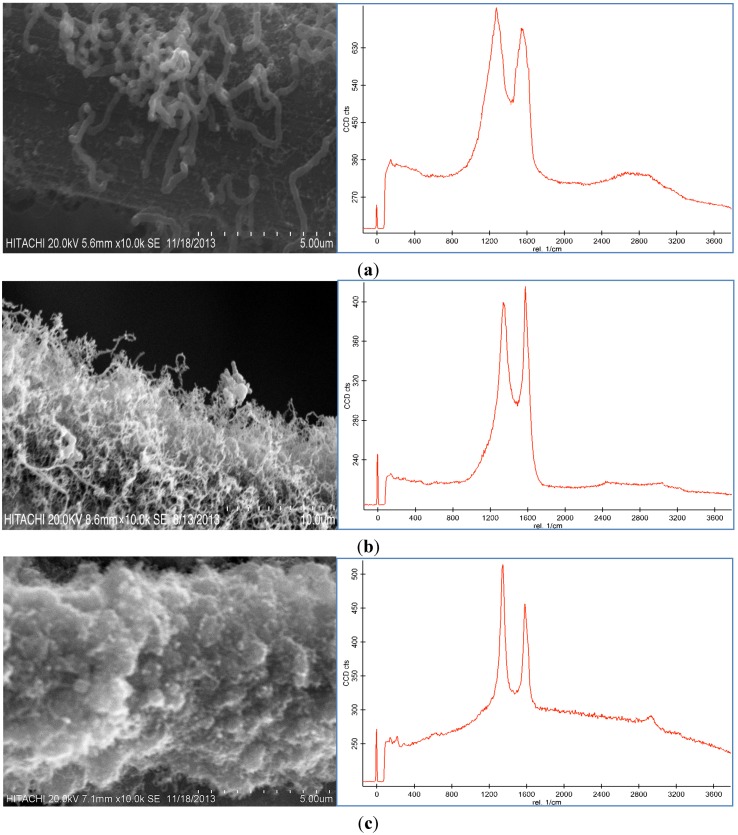
SEM images and Raman spectra of different agglomerations of grown carbon nanofibers (CNFs) on carbon fiber (CF) at (**a**) 50 mM, (**b**) 100 mM, and (**c**) 150 mM catalyst concentrations at 550 °C for 30 min run time under 50 sccm acetylene flow rate.

Representative SEM images and Raman spectroscopy graphs of different forms of the CNF coated CF are shown in Figure 2a–c, and reveal different agglomerations of CNF with different catalyst concentrations. To analyze the effect of the catalyst concentration (50 mM, 100 mM, and 150 mM), reaction temperature at 550 °C, reaction time 30 min, and flow rate of acetylene 50 sccm were fixed and catalyst concentrations were varied.

Based on the SEM images, it was seen that the optimum catalyst concentration was 100 mM as that led not only to high density but also separated the fibers of the CNFs. Using the lower amount of catalyst concentration (100 mM) caused defective growth or insufficient density of the CNF whereas for the greater amounts, it led to thick and loose layers of CNFs on the CF. The Raman Spectra shows two large peaks at 1350 cm^−1^ and 1590 cm^−1^, which were assigned to the D peak from the amorphous carbon structure, and the G peak from the graphitic structures of carbon, respectively [28]. Based on the Raman spectra of the CF it was found that by growing the CNF on the CF surface the graphitization is improved because of the increase of G peak and decrease of D peak. According to the Raman graphs of CF-CNF, it is found that at the 50 mM catalyst concentration, the graph is too wide, which is related to a deficiency of uniform coating of the catalyst layer on the CF surface. At the 150 mM catalyst concentration, the presence of a great number of amorphous carbon structures can be confirmed by the high peak of D. The Raman spectra in Figure 2b illustrate a high graphitization degree of the resultant CNF in comparison with the others.

The BET surface area, thickness, yield of CNFs and activities of the different catalyst concentration on the CF for the CNF growth are presented in Table 1. According to the results from the BET analysis and SEM images, the surface area and thickness of the CF-CNF increased by increasing the catalyst concentration. Increasing the catalyst amount led to an increase in the surface area, thickness, and yield until the formation of the amorphous carbon was established. Consequently, the presence of amorphous carbon at 150 mM as reported in the Raman spectrum concurs with the defects in the surface area. The activity and performance of three catalytic samples (0.5 g CF) in the carbon nanofiber production using CVD are summarized in Table 1.

Table 1 indicates that the surface area, thickness, carbon deposition efficiency (CDE) and catalytic activity for the 150 mM catalyst concentration are the highest but Figure 2c shows that the morphology and graphitization of the resulting CF-CNF is otherwise.

To achieve the optimum parameters, not only the yield and thickness but also the structure and morphology of the CNF are significant. Hence, by increasing the catalyst concentration from 50 mM to 100 mM, the graphitization increases (increased G peak) and the CNF covers the CF surface, completely, and also the yield and thickness consequently increase. On the other hand, by increasing the catalyst concentration to 150 mM, the yield and thickness increase but the presence of amorphous carbon is proven (decreased G peak) that reveals the impurity resulting. Therefore, the 100 mM was selected as an optimum catalyst concentration. Subsequently, based on the structure and morphology, the 100 mM was selected as the optimum concentration with acceptable CDE% and catalyst activity. On the other hand, the outer thickness of CNF on the CF was looser than the inner layer; so by increasing the thickness, the stability of the CNF layer decreased.

**Table 1:** Surface area, thickness, and yield of carbon fiber-carbon nanofiber (CF-CNF) and activities of the different catalyst concentration at 550 °C for 30 min under 50 sccm acetylene flow rate.

Catalyst Conc. (mM)	Surface Area (m^2^/g)	Thickness of Carbon Nanofiber (CNF) (nm)	Yield (%)	Catalyst Activity (g/g)
50	1.36	1500	7	0.65
100	2.31	4000	24	1.6
150	2.52	4500	30	1.81

### 2.3. Effect of Reaction Temperature

**Figure 3:**
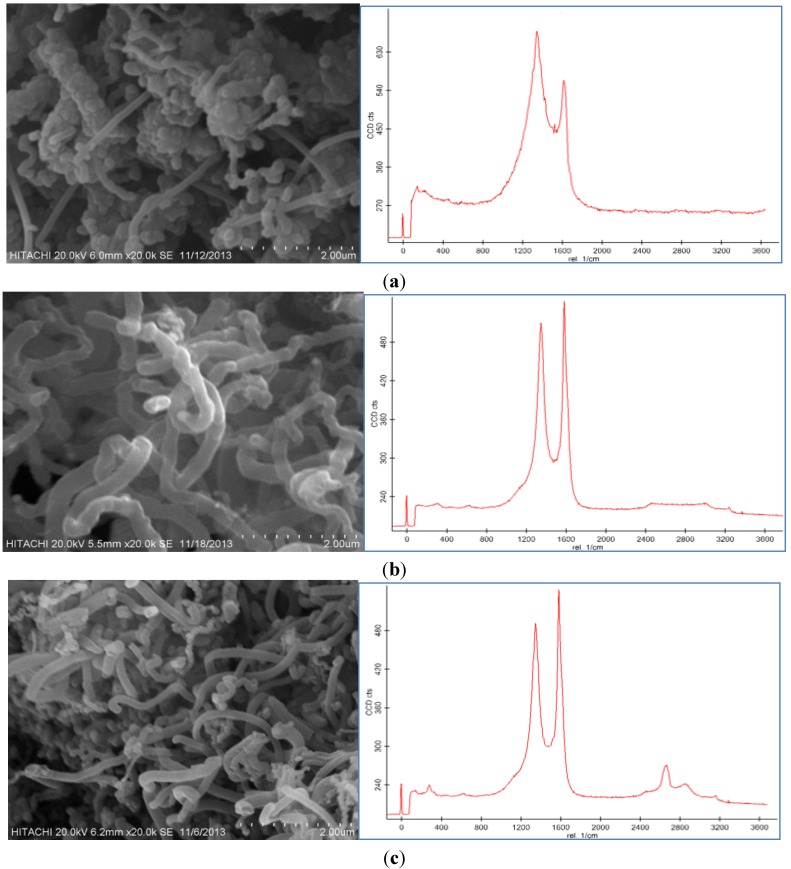
SEM images and Raman spectra of grown CNF by use of 100 mM catalyst concentration for 30 min under 50 sccm acetylene flow rate at (**a**) 450 °C, (**b**) 550 °C, and (**c**) 650 °C.

Three different experiments at various temperatures were performed keeping other parameters including the catalyst concentration of 100 mM, reaction time 30 min, and flow rate of acetylene 50 sccm fixed while the reaction temperature was varied between 450 °C and 650 °C. As can be seen in Figure 3, CNFs were formed on the CF surface at these temperatures. The reaction temperature of the thermal CVD method had a dramatic effect on the CNF growth as shown in Figure 3. It was found that the temperature influenced the morphology and graphitization of the carbon nanoparticles. Based on the Raman spectra, the D peak and G peak at 450 °C were broad, which was possibly due to the presence of amorphous carbon on the CF surface. The D peak was also higher than the G peak. By increasing the temperature, the graphitization of the resulting nanoparticles increased because of the CNF growth (Figure 3b). The Raman spectra in Figure 3c illustrate that the resultant carbon nanoparticle formed at 550 °C exhibited a high degree of graphitization which indicated the presence of carbon nanotubes (CNTs). Therefore, 550 °C was selected as the optimum temperature to grow CNFs of high quality and purity.

Because of the low activity of the catalyst at 450 °C, amorphous carbon formed on the CF, which caused defective structures of the CNFs on the CF (Figure 3a) and decreased the surface area of the CF-CNF (see Table 2). However, by increasing the temperature, the surface area, thickness, and yield of the CF-CNF increased. The highest surface area was obtained at 650 °C, which resulted in the growth of not only CNFs but also CNTs. Besides this, the results indicated that the C_2_H_2_ conversion and carbon yield also increased with increasing reaction temperature. The thickness of the grown CNFs increased slightly by increasing the temperature.

**Table 2:** Surface area and yield of resulting CF-CNF at different growth temperature by use of 100 mM catalyst concentration for 30 min under 50 sccm acetylene flow rate.

Temperature (°C)	BET Surface Area (m^2^/g)	Thickness of CNF (nm)	Yield (%)
450	1.88	3500	13.4
550	2.31	4000	24
650	3.16	4700	32.8

**Figure 4:**
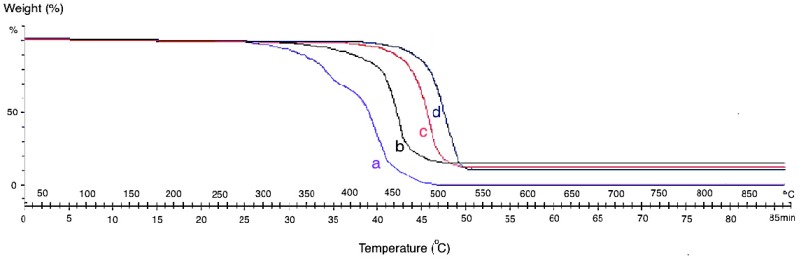
Themogravimetric analysis (TGA) analysis of (**a**) neat CF and CF-CNF at (**b**) 450 °C, (**c**) 550 °C, and (**d**) 650 °C.

The themogravimetric analysis (TGA) of the neat CF and grown CF-CNF at different temperatures is presented in Figure 4. The TGA curve of CF shows that the pyrolytic reactions lead to weight loss starting at about 300 °C for neat CF. By growing CNF on the CF surface, the thermal resistance of the product increases due to the strong structure of the grown CNFs which cause adsorption at the higher temperature.

Since the formation of CNFs on the CF at low temperature was defective so, the formed carbon atoms on the catalyst surface adsorbed the heat only at the low temperature. Therefore, by increasing the reaction temperature, the formation of CNFs was completed and led to the bulk diffusion of absorbed temperature from the adsorbed surface to the growth surface. At 650 °C, significant CNFs and CNTs were already observed. This was due to the aggregation of the catalyst particles and degradation of the carbon source at a higher reaction temperature. However, as it was shown in the SEM images, the best temperature for growing CNFs is 550 °C with a uniform structure, high CDE%, and acceptable thermal stability. The presence of amorphous carbon at 450 °C leads to mass loss sooner than at other temperatures.

### 2.4. Effect of Reaction Time

**Figure 5:**
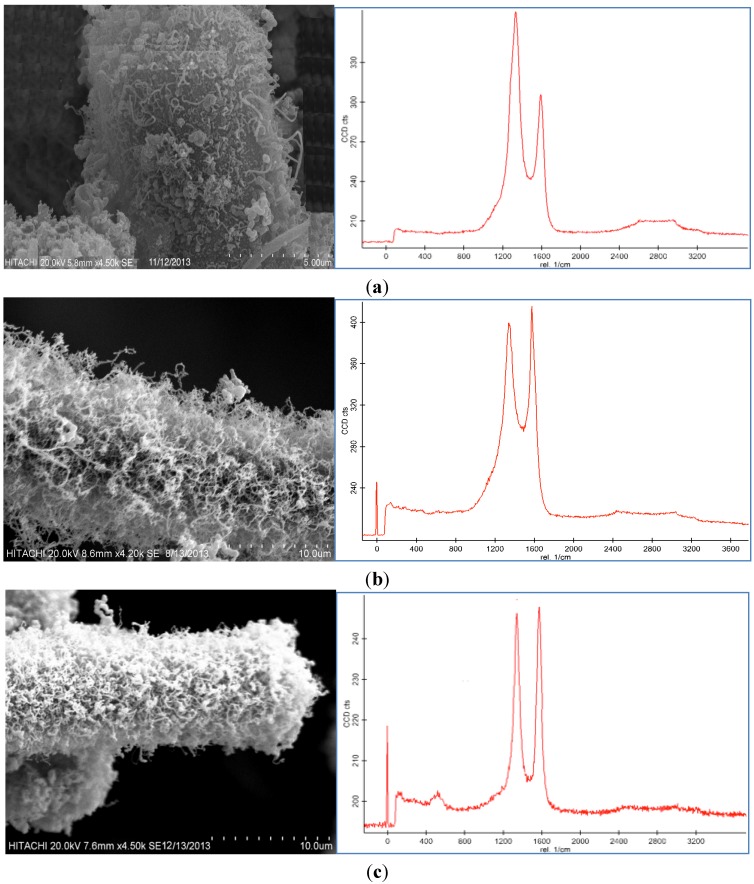
SEM images and Raman Spectrum of CNF morphologies at (**a**) 10 min, (**b**) 30 min, and (**c**) 50 min using 100 mM acid concentration at 550 °C under 50 sccm acetylene flow rate.

Growth time is an alternative parameter, which acts as an important role in dictating the morphology of CNFs. Figure 5a,b displays the SEM images and Raman spectroscopy graphs of the CNF with different growth times (10 and 50 min) by use of the 100 mM catalyst concentration at the 550 °C reaction temperature under a 50 sccm acetylene flow rate. It was observed that different trends occurred at different run times.

According to the SEM micrographs, the synthesis of the CNF at 10 min was too fast to support the proper thickness of the CNF. Therefore, at this run time, CNFs with short fibers were achieved because of the uncompleted formation of the CNFs. Moreover, impurities, such as carbon nanoparticles, amorphous carbon and catalyst particles, were proved from the Raman graph at this run time. The D peak is higher than the G peak and also both are too broad, which affirms the presence of impurities in the sample.

The SEM images of the CNFs at 50 min show an almost similar morphology to the CNF grown at 30 min. The highest thickness and yield of the CNF were obtained at 50 min, however, a relatively thick and high yield CNF was formed at 30 min. The ratio of the G peak to the D peak (*I*_G_/*I*_D_) of the Raman graph in Figure 5b in comparison with Figure 5c implies high graphitization, low amount of amorphous carbon and complete coating of the CNF on the CF surface. At 50 min the intensity of the D peak is similar to the G peak that shows the presence of amorphous carbon and impurity in the product.

The BET surface areas of the different resulting CF-CNFs were calculated from the N_2_ adsorption/desorption isotherms. Table 3 summarizes the data at various stages of preparation. After growing carbon nanofibers on the CF, the surface area increased. The presence of the CNFs with a high BET surface ensures the resulting CF-CNFs have a high surface area. By increasing the growth time to 30 min, the thickness and amount of CNFs on the CF surface was increased, which caused the increase in the overall surface area. Extending the reaction time further to 50 min slowed down the rate of increase in the surface area because of the deactivation of the catalyst particles [29].

Moreover, Table 3 also shows that the yield (CDE%) increased with the reaction time. Running the reaction for 10 min was too short to activate the catalyst particles and to form CNFs to cover the CF completely. Both the CDE% and catalyst activity increased sharply at 30 min. Similarly, extending the reaction time to 50 min only demonstrated a marginal improvement in the CDE% and catalyst activity.

**Table 3:** Effect of growth time on CNF growth by use of 100 mM catalyst concentration at 550 °C under 50 sccm acetylene flow rate (on 0.5 g CF/catalyst, 0.075 g metal weight).

Time (min)	BET Surface Area (m^2^/g)	Thickness of CNF (nm)	Yield (%)
10	1.42	2100	11.6
30	2.31	4000	24
50	2.61	4100	26.2

### 2.5. Effect of Hydrocarbon Flow Rate

**Figure 6:**
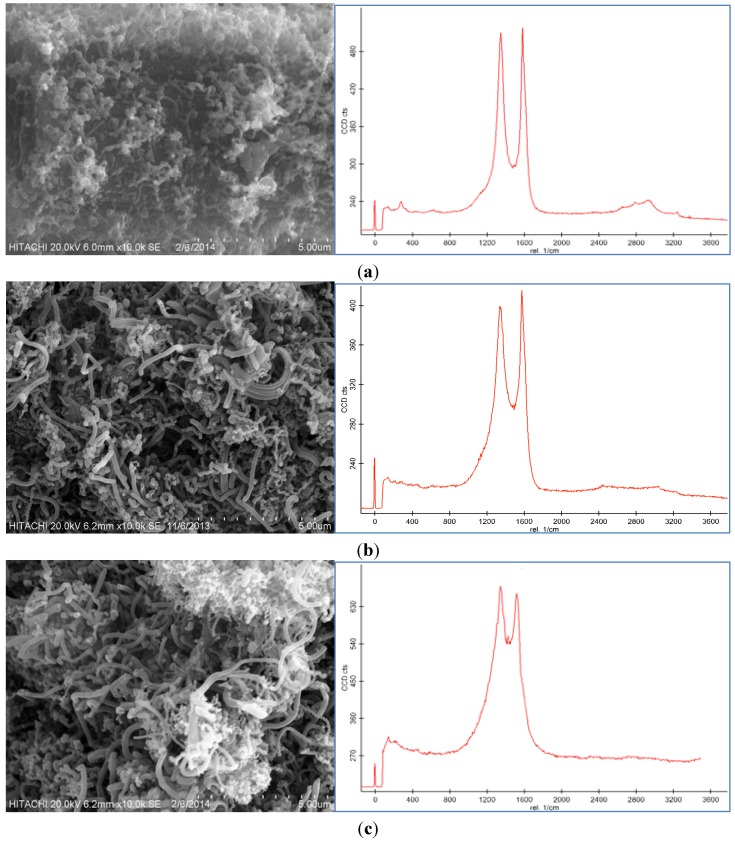
SEM images and Raman spectroscopy of carbon nanofiber on CF by use of 100 mM catalyst concentration at 550 °C for 30 min at (**a**) 25 sccm, (**b**) 50 sccm, and (**c**) 100 sccm flow rate of C_2_H_2_.

Different flow rates led to different structures of the carbon nanofibers; so by changing the carbon source flow rate, the optimum form of CNFs was obtained. The other parameters, such as reaction time (30 min), reaction temperature (550 °C) and catalyst concentration (100 mM) were fixed and the flow rate of the acetylene was altered for 25, 50, and 100 sccm. As can be seen in Figure 6a–c, CNFs were grown on the CF surface, respectively.

The concentration of acetylene influenced significantly the characteristic of the CNFs synthesized by CVD. At 25 sccm, the grown CNFs were in uncompleted fiber form and by increasing the flow rate of the acetylene to 50 sccm, the synthesized CNFs had more regular diameters. The CNFs also had lower amounts of amorphous carbon at the 50 sccm hydrocarbon flow rate than those synthesized at the high flow rate (100 sccm).

At the high acetylene flow rate (100 sccm), the carbon nanoparticles covered all the surface of the CNFs to form a compact coating. During the synthesis of the CNFs, the amorphous carbon nanoparticles nucleated on the external wall of the nanoparticles. The combination of the CNF with the coated amorphous carbon particles led to a broader and longer D peak than the G peak in the Raman spectra.

Consequently, the concentration of acetylene provided another way to control the morphology and graphitization of the synthesized nanoparticles. At a low acetylene flow rate, the acetylene concentration was not sufficient for the synthesis to take place, thus the G peak in the Raman spectra, which was related to the graphitization of the product, was shorter. Thereafter, by increasing the concentration of the hydrocarbon to 50 sccm, the G peak was sharper and longer than the D peak, which revealed the enhanced graphitization and complete formation of the CNFs.

Table 4 lists the BET surface area, thickness, and yield results for the samples. The CF-CNF (50 sccm) had the highest surface area and thickness, implying that it could produce uniform forms of CNFs on the CF. Hence, by changing the flow rate of the carbon source (acetylene) on the CF surface, the surface area and thickness of the resulting CF-CNF changed, 50 sccm > 100 sccm > 25 sccm. In addition, the yield of the CNFs was also affected by the acetylene flow rate. Table 4 reveals that the yield of the CNF increased with increasing the carbon source flow rate. Approximately 0.093 g of CNF was produced when the acetylene flow rate was set at 25 sccm, and 0.12 g and 0.144 g CNF were produced at 50 sccm and 100 sccm of the acetylene flow rate, respectively. However, the amount of soot rose immediately as the carbon source flow rate was increased.

**Figure 7:**
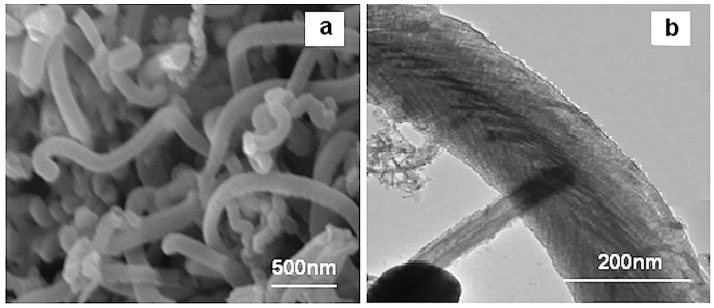
(**a**) SEM and (**b**) transmission electron microscopy (TEM) micrographs of optimum CNF.

In conclusion, the optimum growth of the CNFs on the CF were obtained by using the 100 mM catalyst concentration and 50 sccm acetylene flow rate in the CVD method at 550 °C for 30 min. The morphology of the optimum CNF was analyzed by SEM and Transmission electron microscopy (TEM) which is presented in Figure 7a,b, respectively. As observed from these images, the CNFs consisted of fibers with diameters of about 100–250 nm.

**Table 4:** Effect of hydrocarbon flow rate on CNF growth by use of 100 mM catalyst concentration under 550 °C for 30 min (on 0.5 g CF).

Flow Rate (sccm)	BET Surface Area (m^2^/g)	Thickness of CNF (nm)	Yield (%)
25	1.37	1800	18.6
50	2.31	4000	24
100	2.12	3800	28.8

In summary, the result shows that the thickness, surface area, and yield of the samples increased as the catalyst concentration, reaction temperature, CNF growth time and hydrocarbon flow rate increased. On the other hand, a higher thickness made a looser layer of CNF, which detached from the CF surface easily, and this claim could be proved by the analysis of the mechanical test of this filler in the polymer matrix. Therefore, in order to find an optimum thickness, surface area, and amount of CNF on the CF, mechanical tests should be carried out and analyzed.

One of the scopes of this research is related to improving, the polymer composite using CNF-coated CF. An important factor in achieving this scope of the study is having the proper thickness, surface area and also amount of uniform CNFs on the CF.

### 2.6. Mechanical Properties

CNFs and CFs with high aspect ratios, low weight and high tensile strength are expected to be used as nanofillers in the polymers to prepare the composites due to their special properties (e.g., CNFs’ Young’s modulus = 500 GPa and CFs’ = 231 GPa) [30,31,32]. The optimum form of the CNF coated CF causes not only an improvement in the fiber-matrix tensile properties but also has synergic effects as a reinforcing factor.

In Table 5, the influences of the different thicknesses of the CNF including low thickness (CNF_L_), medium thickness (CNF_M_) and high thickness (CNF_H_) on the surface area and also in the polymer composites on the tensile stress and tensile modulus of the composites were analyzed. The improvement of the tensile stress of the CF-CNF_H_/PP composite compared to the CF-CNF_L_/PP composite confirms the significant enhancement in the mechanical properties of the CF-CNF_H_/PP composite.

**Table 5:** Tensile data for different thickness of CNF in CF-CNF/PP composite.

Sample No.	Thickness (nm)	Surface Area (m^2^/g)	Tensile Stress (MPa)	Tensile Modulus (GPa)
CF-CNF_L_/PP	1500–2100	1.36–1.42	21.9–22.2	0.65–0.68
CF-CNF_M_/PP	3500–4000	1.88–2.31	22.7–23.1	0.70–0.73
CF-CNF_H_/PP	4100–4700	2.61–3.16	23.9– 24.8	0.75–0.79

Regarding the comparison of the composite stiffness fabricated with the different thicknesses and surface areas of the CNF, it reveals an improvement in the tensile modulus and stress of the CF-CNF_H_/PP composite. The reduction of the tensile stress and Young’s modulus of the composite was related to the defective flow of the matrix around the thin CNF layer on the CF in the polymer matrix which led to the decrease of the interfacial properties and it being easily pulled out of the CF from the polymer matrix [30]. Similarly, the strength of the CF-CNF_H_/PP composite was higher than the CF-CNF_L_/PP composite because of the high stress transfer between the CNF_H_ and the matrix [33]. Such a phenomenon can be observed from the tensile tests of different forms of the CF-CNF composites.

The relationship between the mechanical properties of the composites and the reinforcement fillers has been systematically investigated. Mathematical models were used to predict the mechanical properties of the different composites. The Halpin–Tsai (HT) Equation (1) is an accepted and extensively adopted model to calculate the stiffness of fiber/polymer composites [27]. The HT model correlated the stiffness of the composites with the tensile modulus of the matrix and the reinforcement as well as their volume contents and geometries. This model was implemented to predict the tensile modulus of the composites with unidirectional or randomly distributed fibers.

In this calculation, different thicknesses of the CNF layers were assumed as fillers with random distributions in the polypropylene matrix. By thinking about the incorporation of the three types of reinforcements (CNF_L_, CNF_M_, and CNF_H_) within the matrix, the HT equations were modified according to the following equation (Equation (1)) [34]:

(1) $$E_{c}=\;\frac{3}{8}V_{f}E_{f}+\;\frac{5}{8}V_{m\;}E_{m}$$

where *E_c_* was the modulus of the composite, *E_f_* was the Young’s modulus of the filler, *V_f_* was the filling content of the filler, *E_m_* was the Young’s modulus of the polymer matrix, and *V_m_* was the filling content of the polymer matrix.

Afterwards, the effective reinforcement modulus of the fillers (Equation (2)) was obtained as follows:

(2) $$E_{f}=\frac{E_{c\;}–\;\frac{5}{8}V_{m}E_{m}}{\frac{3}{8}V_{f}}$$

**Figure 8:**
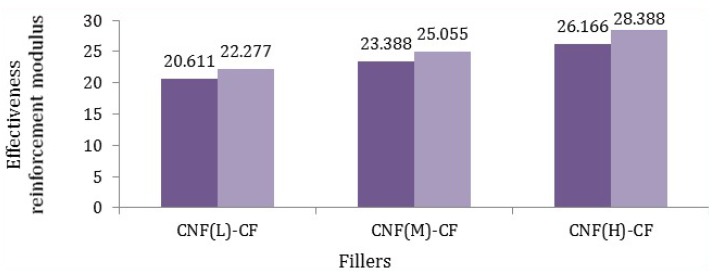
Effective reinforcement modulus of different fillers in polypropylene matrix (dark purple states minimum amount and light purple reveals maximum amount).

Based on Equation (2), the Young’s modulus of various fillers was calculated and is reported in Figure 8, where *E_c_* was obtained from Table 5, *E_m_* was about 0.47 GPa, *V_f_* was 5%, and *V_m_* was 95%.

According to Figure 8, it was found that the modulus of fibers (CF-CNF_H_) was the highest. Such a meaningful difference was related to not only the uniform and excellent coating of the CNF on the CF but also the maximum surface area of the CNF. By comparing the Young’s modulus of fibers, it can be calculated that the reaction time and catalyst concentration have the main roles rather than temperature and hydrocarbon flow rate, which was verified by comparing the tensile modulus of the different CNF-CFs. Consequently, the mathematical calculations confirm the experimental results of the tensile modulus of fillers.

### 2.7. Morphology of Composites

**Figure 9:**
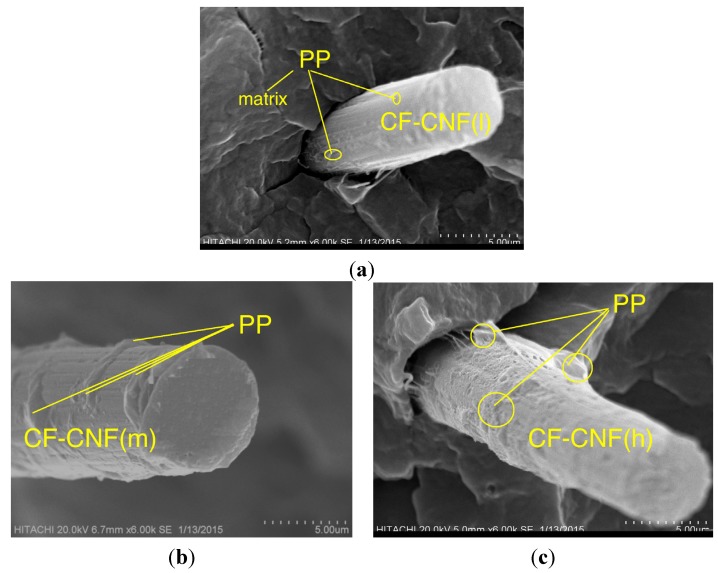
SEM micrographs of fractured surface of (**a**) CF-CNF_L_/PP, (**b**) CF-CNF_M_/PP, and (**c**) CF-CNF_H_/PP composites.

The SEM micrographs in Figure 9 illustrate the fractured surface of the different CF-CNF/PP composites. Figure 9a shows that the CF-CNF_L_/PP has the minimal interfacing of the CF surface with the matrix because of the smooth surface of the CF. On the contrary, the presence of CNF_M_ as the rough coated phase on the CF acted against the smooth neat CF as is shown in Figure 9b. The CF-CNF_H_/PP was revealed in Figure 9c. The presence of the PP residue on the CF-CNF_H_ surface confirms the enhancement of the adhesion between the fiber and PP matrix due to the powerful interlocking between them.

## 3. Experimental Section

### 3.1. Materials

High purity acetylene (C_2_H_2_) as a carbon source gas, nitrogen (Air Product, 99.9995%) as a carrier gas, nickel nitrate hexahydrate (Ni(NO_3_)_2_·6H_2_O) as a catalyst source and chopped-unsized carbon fiber (CF) (100 μm) as a substrate were used in the experimental part of this research. Polypropylene pellets (PP 600G) were utilized as a polymer matrix purchased from Petronas Polymer Marketing and Trading Division, Kuala Lumpur, Malaysia.

### 3.2. Synthesis of CNFs on CF

To obtain different surface areas, thicknesses, and yields of CNFs, the effective parameters in this process, such as catalyst concentration, reaction temperature, time, and acetylene flow rate, were varied. Table 6 describes the fixed and changeable parameters during the CNF growth on CF by the CVD process. Firstly, the chopped carbon fibers were immersed into nickel nitrate hexahydrate solution with different concentrations (50 mM, 100 mM, 150 mM) and agitated by ultrasonic agitation for 2 h. Then, they were dried and calcinated at 200 °C under airflow to eliminate nitrate components in order to achieve the catalyst coating on the surface of the CF. The CVD method was applied to grow the CNFs on the CF under atmospheric pressure at temperatures from 550 to 650 °C for 10–50 min. This process was fulfilled by the catalytic reaction of acetylene (25, 50, and 100 sccm) over a Ni/CF surface in the reactor under a flow rate of H_2_/N_2_ (100, 100 sccm). At the end of the run time, the C_2_H_2_ flow was stopped, the heater was turned off and then, the reactor was cooled under N_2_ flow. Investigation of the surfaced morphology and structural information of the product were inspected through the electron microscopes (SEM and TEM), BET surface area, and Raman spectroscopy.

The scanning electron microscope (SEM) (mode, Model: NOVA NANOSEM 230, Voltage: 1 kV–30 kV, SE detector: TLD, BSE detector: Low kV SSBSED) (FEI Company, Hillsboro, OR, USA) is a type of electron microscope that detects signals from the interaction of the incident electrons with surface of the samples by focusing a high-energy beam of electrons on the sample surface. Transmission Electron Microscopy (TEM) HITACHI-7100 (HITACHI Limited, Tokyo, Japan) was used to observe clearly the carbon nanoparticles on the CF surface. The Brunauer–Emmett–Teller (BET) technique was employed to analyze the specific surface area of the resulting samples based on the ISO 9277. Raman spectroscopy (alpha 300 R, WITec, Ulm, Germany), which is one of the most sensitive methods for studying carbon materials, provides very important information on the microstructure and crystalline order of carbon materials.

**Table 6:** Different operating conditions for different CNF growth.

Step	Fixed Parameters	Variable Parameters
1	Temperature: 550 °C Time: 30 min Flow rate of C_2_H_2_: 50 sccm	Catalyst Concentration:
		50 mM
		100 mM
		150 mM
2	Catalyst Concentration: 100 mM Time: 30 min Flow rate of C_2_H_2_: 50 sccm	Temperature:
		450 °C
		550 °C
		650 °C
3	Catalyst Concentration: 100 mM Temperature: 600 °C Flow rate of C_2_H_2_: 50 sccm	Time:
		10 min
		30 min
		50 min
4	Catalyst Concentration: 100 mM Temperature: 600 °C Time: 30 min	Flow rate of C_2_H_2_:
		25 sccm
		50 sccm
		100 sccm

### 3.3. Carbon Deposition Efficiency (Yield)

The process efficiency was characterized through the weight of the deposited carbon, during each run. The carbon deposition efficiency (CDE), corresponding to the percentage of the deposited carbon in comparison with the introduced quantity of the carbon fiber coated with catalytic particles was calculated as below (Equation (3)):

(3) $$\mathrm{CDE}\%=\frac{\mathrm{PM}-\mathrm{CM}}{\mathrm{CM}}\times 100$$

where PM was the mass of the product and CM was the mass of the initial carbon fiber coated with the catalyst.

### 3.4. Catalyst Activity Calculations

Catalyst activity (CA) is basically defined as the ratio of the mass product to the metal mass (Equation (4)).

(4) $$\mathrm{CA}=\frac{\mathrm{PM}-\mathrm{CM}}{\mathrm{MM}}$$

where MM was the mass of the transition metals, which coated on the CF surface.

### 3.5. Composites Preparation

To compose the composite nanomaterial, the PP was melted and blended in a mixer (Thermo Haake Poly Drive R600/610) (LabX, Midland, Canada) at 180 °C with a 55 rpm rotor speed for 5 min and then mixed with fibers (5 wt %) and blended for 15 min [35]. The resultant blended and melted composite was put in a mold of the size 15 cm × 15 cm with a 1 mm thickness, allowed to melt at 180 °C under a pressure of 150 kg/cm^2^ by HSINCHU Hot Press Machine (Tradekey, Taiwan), and then cooled to 60 °C.

### 3.6. Composite Characterization

The composite was cut by the ASTM D638 standard using dumbbell-shaped bars with a thickness of 1 mm [36]. The tensile test was performed by using an Instron Universal Testing Machine (Instron, Canton, MA, USA) at room temperature to measure the tensile modulus and stress of the PP, CF/PP, and CF-CNF/PP. The tests were carried out with a crosshead speed of 5 mm/min [37]. Besides this, the fractured surface of the composites was analyzed by SEM images, which state the interaction of the filler with the polymer matrix.

## 4. Conclusions

The most important scope of this research is concerned with the study of the influences of the thickness and surface area of the CNF layer coated CF on the enhancement of the PP composite. Therefore, the surfaces of the CF with CNF layers were modified by using CVD using different concentrations of the catalyst, from 50 mM to 150 mM at various reaction temperatures, from 450 to 650 °C, at different run times of 10 to 50 min, and under 25–100 sccm acetylene flow rates. The evidence of an intensively CNF coated CF at 550 °C using the 100 mM catalyst concentration under the 50 sccm acetylene flow rate at 50 min was demonstrated by SEM and TEM micrograph images, BET surface area, and Raman spectroscopy graphs. Apart from the thickness, surface area, and yield, other properties such as graphitization, structure and morphology of the product are important to find the optimum conditions. Considering the results, it can be seen that by increasing the catalyst concentration to 100 mM, temperature to 550 °C, run time to 30 min, and acetylene flow rate to 50 sccm, the yield, thickness and surface area increase and also graphitization and purity are improved. Conversely, by increasing the parameters to 150 mM for catalyst concentration, 650 °C for temperature, 50 min for run time, and 100 sccm for acetylene flow rate, the yield, thickness, and surface area increased but the graphitization and purity dropped. Besides this, effective reinforcement was predicted by the mathematical model and it was found that the thickness of the CNF had the main role, which was verified by comparing the tensile modulus of the different fillers. It can be deduced that CNF_H_ with the highest surface area and thickness acts as a reinforcement which leads to the enhancement in the mechanical properties of the CF composites.
